# Resting-state networks representation of the global phenomena

**DOI:** 10.3389/fnins.2023.1220848

**Published:** 2023-08-17

**Authors:** Shiori Amemiya, Hidemasa Takao, Shouhei Hanaoka, Osamu Abe

**Affiliations:** Department of Radiology, Graduate School of Medicine, University of Tokyo, Tokyo, Japan

**Keywords:** resting-state functional magnetic resonance imaging, hemodynamic response function, cerebrovascular perfusion, blood oxygenation level-dependency, neurovascular coupling

## Abstract

Resting-state functional magnetic resonance imaging (rsfMRI) has been widely applied to investigate spontaneous neural activity, often based on its macroscopic organization that is termed resting-state networks (RSNs). Although the neurophysiological mechanisms underlying the RSN organization remain largely unknown, accumulating evidence points to a substantial contribution from the global signals to their structured synchronization. This study further explored the phenomenon by taking advantage of the inter- and intra-subject variations of the time delay and correlation coefficient of the signal timeseries in each region using the global mean signal as the reference signal. Consistent with the hypothesis based on the empirical and theoretical findings, the time lag and correlation, which have consistently been proven to represent local hemodynamic status, were shown to organize networks equivalent to RSNs. The results not only provide further evidence that the local hemodynamic status could be the direct source of the RSNs’ spatial patterns but also explain how the regional variations in the hemodynamics, combined with the changes in the global events’ power spectrum, lead to the observations. While the findings pose challenges to interpretations of rsfMRI studies, they further support the view that rsfMRI can offer detailed information related to global neurophysiological phenomena as well as local hemodynamics that would have great potential as biomarkers.

## 1. Introduction

Resting-state functional magnetic resonance imaging (rsfMRI) is a method of investigating spontaneous neural activity, often based on its macroscopic organization characterized by the coherence of the activity. The spatial patterns identified as areas with synchronous oscillation of the blood oxygenation level–dependent (BOLD) signal are termed resting-state networks (RSNs; Fox et al., 2005). These networks are closely related to the anatomical connectivity among the neural subsystems that have been revealed by a wide variety of visual, sensorimotor, and cognitive task paradigms (Vincent et al., 2007; Zhang et al., 2010). However, the neurophysiological mechanism underlying the synchronization within the RSNs remains to be elucidated.

On the other hand, accumulating evidence points to a substantial contribution from the global physiological signals to the generation of structured synchronization within the RSNs (Amemiya et al., 2023); Tong et al. were the first to show that the time lag or delay of the low-frequency fMRI signal can give rise to spatial patterns similar to those of the RSNs (Tong et al., 2015). The time lag was computed voxel-wise by employing a data-driven recursive approach applied to the fMRI data or by setting the middle finger vascular signal as the reference signal. Such extracranial signals measured with near-infrared spectroscopy have been shown to correlate well with the signal from the cerebrovascular system (Tong and Frederick, 2010, 2012, 2014a,b; Tong et al., 2011, 2012, 2013, 2019; Tong and Hocke, 2014). An equivalent time lag map can be obtained by setting the global mean signal as the reference signal, which has been shown to share similar spatiotemporal characteristics with cerebrovascular perfusion (Lv et al., 2013; Amemiya et al., 2014, 2016, 2022; Tong et al., 2017) or hemodynamic responses to an acetazolamide challenge (Nishida et al., 2019) and a carbon dioxide challenge (Yao et al., 2021). The contribution of the time lag to the generation of RSN synchronization was also confirmed in our study examining the spatiotemporal characteristics of the resting-state global signals identified by using temporal independent component analysis (ICA; Amemiya et al., 2019). More recently, Chen et al. also showed that regional variability of the hemodynamic response functions to physiological events such as respiration and heartbeat leads to the generation of the “physiological networks” corresponding to those of the RSNs (Chen et al., 2020). All these findings consistently support the view that the global physiological events triggering the hemodynamic responses can cause local networks similar to the RSNs. In line with these studies, we also confirmed that the regional variation of the signal time lag patterns are similar regardless of whether the assumed source of the component is physiological events (global mean signal), spontaneous neural activity (RSN signals), or simultaneous neural stimulation (visual task fMRI signals; Amemiya et al., 2020). Such observations indicating that the regional variation of the hemodynamics is a non-specific source of a similar time lag pattern in fMRI lead to a corollary question regarding the specificity of the source of each RSN signal component. More specifically, seemingly independent RSN signals that are supposed to reflect local neural activity specific to each network might be distilled into a single set of global phenomena.

In relation to this point, several previous studies have explored the spatiotemporal characteristics of the rsfMRI signals by examining or decomposing the global rsfMRI signal time lag (Mitra et al., 2015; Amemiya et al., 2016; Bolt et al., 2022). The use of the time lag has advantages in rsfMRI studies, in which it is difficult to know the time course of the neural activity or to extract precise hemodynamic response functions to neural events in each region. Even in such a case, time lag analysis can provide information if we can assume a common condition for either the stimuli or hemodynamic response function. However, the factors affecting the signal time lag in rsfMRI studies have not been fully examined in previous studies, leaving room for ambiguity about the complete picture of what is happening in the resting brain.

This study aims to address these issues by examining the spatiotemporal patterns of the rsfMRI global signal component, taking advantage of the inter- and intra-individual variation of the signal time delay. We hypothesized that such measurements add complemental information as to the spatiotemporal pattern of the rsfMRI signals to the previous studies that are based on the average time lag or the average hemodynamic response functions. The theoretical rationale was that the hemodynamics or the hemodynamic response functions are empirically known to be non-homogenous across the brain or the subjects and are non-stationary over time. Regional variations of the vascular architecture or anatomy that determine the diameter and density of the local arterioles and capillaries (Harrison et al., 2002; Weber et al., 2008; Kim and Ogawa, 2012) or the duration and intensity of the stimulus triggering the arterial dilation (Logothetis et al., 2001; Martindale et al., 2005; Kennerley et al., 2012; Martin et al., 2013) all affect the hemodynamic response function. These variations giving rise to the inter- and intra-subjects variability of the hemodynamic response function (Aguirre et al., 1998; Handwerker et al., 2004; Amemiya et al., 2012) might explain why some RSNs do not manifest as a single network even though their hemodynamic responses are supposed to be overlapping based on their (for example, symmetric) anatomical location. Another point was that, as we will show in the theory section, the regional variability of the hemodynamic response function, combined with the temporal changes in a shared stimulus function, can theoretically give rise to variations in the signal time lag. Therefore, even if the hemodynamic response functions were stable across time in each region, the regional variation of the hemodynamic response function can theoretically lead to temporal changes in the time lag along with the temporal changes in the shared stimulus function, thereby directly affecting the spatiotemporal patterns and its dimensionality of the rsfMRI signal induced by a single set of global phenomena.

To test these hypotheses, we examined the spatial patterns of the inter- and intra-subject variability of the time lag computed voxel-wise by setting the global mean signal as the reference signal. For the intra-subject variability, multiple time lag maps were computed for each subject by using a sliding window approach. Since temporal correlation is highly sensitive to the signal time lag and signal-to-noise ratio, in addition to the time lag, it has also been proven to be a good marker of hemodynamic changes in patients undergoing revascularization surgery (Amemiya et al., 2022). Therefore, in addition to the time lag, maps of the correlation coefficients between the global mean signal and each voxel’s signal were also subjected to the same analyses.

## 2. Theory

In this section, we describe how the time lag Δ*t* that minimizes the Euclidean distance between the two time series X(t) and $X^{\prime }\left(t\right)$ that are generated by the same stimulus function u(t) convolved with the different hemodynamic response functions h(t) and $h^{\prime }\left(t\right)\;$ changes depending on the stimulus function and response functions.

Assuming that the BOLD signal is the output of a linear time-invariant system (Boynton et al., 1996), we can express the expected signal X(t) and $X^{\prime }\left(t\right)$ with the convolution operator ⊗ as follows:


(1) $$X\left(t\right)≜\left\{u⊗h\right\}\left(t\right)=\overset{\infty }{\underset{-\infty }{\int }}u\left(\tau \right)\;h\left(t-\tau \right)d\tau$$

(2) $$\begin{array}{c} X^{\prime }\left(t\right)≜\left\{u⊗h^{\prime }\right\}\left(t\right)=\overset{\infty }{\underset{-\infty }{\int }}u\left(\tau \right)\;h^{\prime }\left(t-\tau \right)d\tau \end{array}$$

Then, the difference between Eq. (1) and (2), with the latter lagged by Δ*t*, is described as follows:


(3) $$\begin{array}{c} \begin{array}{l} d\left(t\right)≜X^{\prime }\left(t-\Delta t\right)-X\left(t\right)=\\ \;\;\;\;\;\;\;\;\;\;\;\;\;\;\;\;\;\overset{\infty }{\underset{-\infty }{\int }}u\left(\tau \right)\;\{h^{\prime }\left(t-\Delta t-\tau \right)-h\left(t-\tau \right)\}d\tau \end{array} \end{array}$$

If we define the difference between the two response functions as follows:


(4) $$r\left(t\right)≜h^{\prime }\left(t-\Delta t\right)\Delta t-h\left(t\right)$$

then, Eq. (3) is described as follows:


(5) $$\begin{array}{c} \;d\left(t\right)=\overset{\infty }{\underset{-\infty }{\int }}u\left(\tau \right)\;r\left(t-\tau \right)d\tau =\left\{u⊗r\right\}\left(t\right) \end{array}$$

By taking the integral of the square of Eq. (5), we have


(6) $$\begin{array}{c} \overset{\infty }{\underset{-\infty }{\int }}{\left|d\left(t\right)\right|}^{2}dt=\overset{\infty }{\underset{-\infty }{\int }}{\left|\left\{u⊗r\right\}\left(t\right)\right|}^{2}dt \end{array}$$

Let U , R , and D be the Fourier transform F of u , r , and d , respectively, as follows:


(7) $$\begin{array}{c} U\left(f\right)≜F\left\{u\right\}\left(f\right) \end{array}$$

(8) $$\begin{array}{c} R\left(f\right)≜F\left\{r\right\}\left(f\right) \end{array}$$

(9) $$\begin{array}{c} D\left(f\right)≜F\left\{d\right\}\left(f\right) \end{array}$$

According to the convolution theorem,


(10) $$\begin{array}{c} F\left[\left\{u⊗r\right\}\left(t\right)\right]=U\left(f\right)\;\;.\;\;R\left(f\right) \end{array}$$

From Eqs. (5), (9), and (10), we derive


(11) $$\begin{array}{c} D\left(f\right)=U\left(f\right)\;\;.\;R\left(f\right) \end{array}$$

Using Parseval’s theorem,


(12) $$\begin{array}{c} \overset{\infty }{\underset{-\infty }{\int }}{\left|d\left(t\right)\right|}^{2}dt=\overset{\infty }{\underset{-\infty }{\int }}{\left|D\left(2\pi f\right)\right|}^{2}df \end{array}$$

From Eqs. (11) and (12), we have


(13) $$\begin{array}{c} \overset{\infty }{\underset{-\infty }{\int }}{\left|d\left(t\right)\right|}^{2}dt=\overset{\infty }{\underset{-\infty }{\int }}{\left|U\left(2\pi f\right)\;\;.\;\;R\left(2\pi f\right)\right|}^{2}df \end{array}$$

This is transformed into


(14) $$\begin{array}{c} \overset{\infty }{\underset{-\infty }{\int }}{\left|d\left(t\right)\right|}^{2}dt=\overset{\infty }{\underset{-\infty }{\int }}{\left|U\left(2\pi f\right)\right|}^{2}\;\;.\;\;\;{\left|R\left(2\pi f\right)\right|}^{2}df \end{array}$$

Therefore, the time lag Δ*t* that minimizes the difference between the two time series varies depending on the integral of the product of the power spectrum of the stimulus function u(t) and that of the difference of the response functions h(t) and $h^{\prime }\left(t\right)$ .

## 3. Materials and methods

### 3.1. Dataset

The dataset was originally from the WU-Minn human connectome project (HCP) young healthy adults (ages 22–35) S1200 release^1^ acquired over consecutive days (day 1 and 2), which is the same as the one we used in our previous studies (Amemiya et al., 2019, 2020; Amemiya et al., 2022). A total of 200 runs from 50 subjects (50 subjects × 2 phase-encoding directions × 2 days, 32 women; average age: 29.4 ± 3.3 years) who underwent 15-min 3.0-T rsfMRI sessions (with a repetition time [TR] of 0.72 s, 1,200 volumes/run) without quality control issues, whose mean framewise displacement was less than 0.2 mm, which had been minimally processed, (Glasser et al., 2013) were subjected to the following analyses.

### 3.2. Time delay and correlation maps

**Figure 1:**
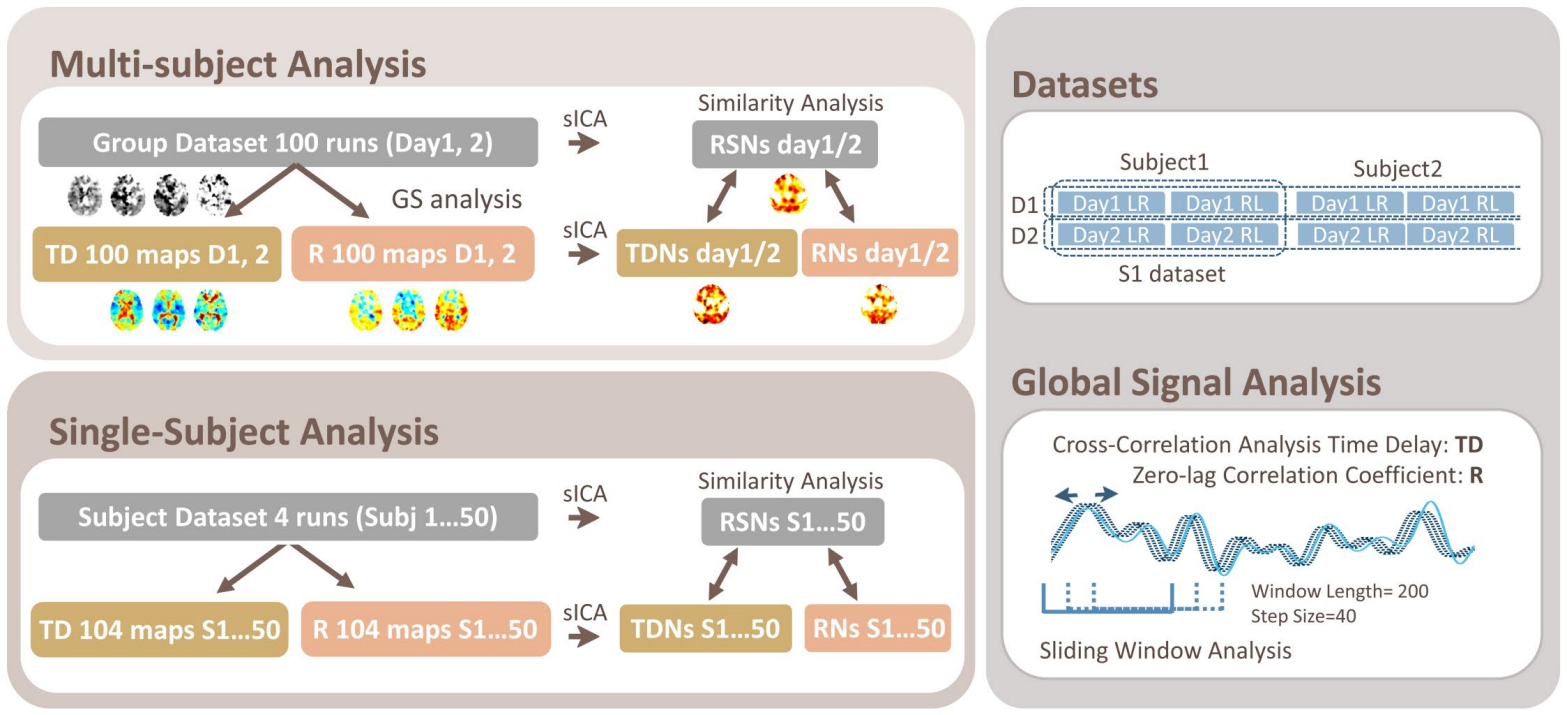
Schematic of the Data Analysis. In both the multi-subject and single-subject analyses, cross-correlation analysis was applied to the preprocessed rsfMRI data to compute the time delay (TD), giving the maximum correlation coefficient (R) voxel-wise by setting the global mean signal as the reference. For the single-subject analysis, a sliding window approach was used to acquire 26 TD and R maps for a run. The multiple TD and R maps for each group (Day 1 and Day 2 or D1 and D2) or for each subject (Subj 1…50 or S1…S50) were subjected to the spatial independent component analysis (sICA) to acquire TD and R networks (TDNs and RNs), respectively. The similarity between the TDNs/RNs and the resting-state network (RSN) maps acquired by directly applying ICA to the corresponding original dataset was compared within the gray matter.

The time delay (TD) and temporal correlation (Pearson’s correlation coefficient: R) were computed for each voxel by setting the global mean signal (average whole-brain gray matter signal time series) as the reference signal as in previous studies (Amemiya et al., 2014, 2016, 2019, 2020, 2022; Figure 1); briefly, after spatial smoothing with a Gaussian kernel with a full-width at half-maximum of 8 mm, linear trend removal, and band-pass filtering at 0.01–0.1 Hz, TD was computed as the relative time lag *t* that gives the best positive fit between each voxel’s time series and the time-shifted (± 6 s or ±8.3 TR) reference signal using cross-correlation analysis (Amemiya et al., 2014, 2016, 2019, 2020). All data were up-sampled to a resolution of 0.14 s (1/5 TR) for the analysis (Tong et al., 2017). For intra-subject variability, multiple TD and R maps were acquired using a sliding-window approach (window length/step size = 200/40 volumes, 26 maps/run).

### 3.3. ICA of time delay and correlation maps

Spatial ICAs were performed on multi-subject and single-subject datasets, respectively, to extract the local hemodynamic networks represented in the TD and R maps. ICA assumes that observed data $x_{i},i=1,..,n$ can be modeled as linear combinations of independent components $s_{j},j=1,..,m$ with some unknown coefficients $a_{ij}$ , which can be described as:


$$\begin{array}{c} x=A\;.\;s \end{array}$$

where A represents the mixing matrix that collects the coefficients $a_{ij}$ (Hyvärinen, 1999). In this case, $x_{i}$ corresponds to the measured TD and R maps, and i=1,..,n are different observations (runs, subjects, and sliding windows), whereas $s_{j}$ corresponds to estimated hemodynamic networks and $a_{ij}$ represents the mixing weight.

The networks were obtained by using the multi-subject datasets, each of which was composed of 100 maps of TD and R (50 subjects x 2 phase-encoding directions) that were subjected to spatial ICA using Multivariate Exploratory Linear Optimized Decomposition into Independent Components (MELODIC) 3.15 (Beckmann and Smith, 2004), part of FMRIB’s Software Library (FSL; https://www.fmrib.ox.ac.uk/fsl) with the dimensionality set to 30, respectively. The networks were also obtained for each subject using 104 TD and R maps (26 maps × 4 runs) that were similarly concatenated and subjected to spatial ICA with the dimensionality set to 30, respectively.

### 3.4. Resting state networks

A conventional multi-session temporal concatenation multi-subject spatial ICA was performed on each of the corresponding preprocessed datasets. The dimensionality was set to 30. For each dataset, template matching was performed to identify 10 independent components (ICs) that best correlate with 10 RSN templates (Smith et al., 2009). The templates are considered the major representative functional networks corresponding to the medial, lateral, and occipital visual areas, default mode network (visuospatial system), auditory system, sensory-motor system, frontoparietal networks (dorsal visual stream), executive control, and cerebellum, respectively (Beckmann et al., 2005; Smith et al., 2009). These RSNs were compared with the ICA components obtained by decomposing the TD and R maps to examine if RSNs are represented as the networks of hemodynamics.

### 3.5. Statistical analyses

The similarity between the maps was evaluated with Pearson’s correlation coefficients within a gray matter mask that was created by thresholding the gray matter tissue map at a ≥30% probability of being gray matter. Statistical analyses were performed using MATLAB version 9.12.0 (MathWorks, Natick, MA). *p* values less than 0.05 were considered statistically significant. Correlation coefficients were Fisher’s *Z* transformed to be subjected to a one-sample t-test against the null hypothesis of no correlation. In addition to the main ICAs, we also examined the inter- and intra-subject standard deviations of the TD and R to infer the source of the variations. The effect of motion on the variability of the TD and R measurement for each RSN was also examined for each run, using the mean frame-wise displacement that was computed following (Power et al., 2012).

## 4. Results

### 4.1. Variability of The time delay and correlation measurements

**Figure 2:**
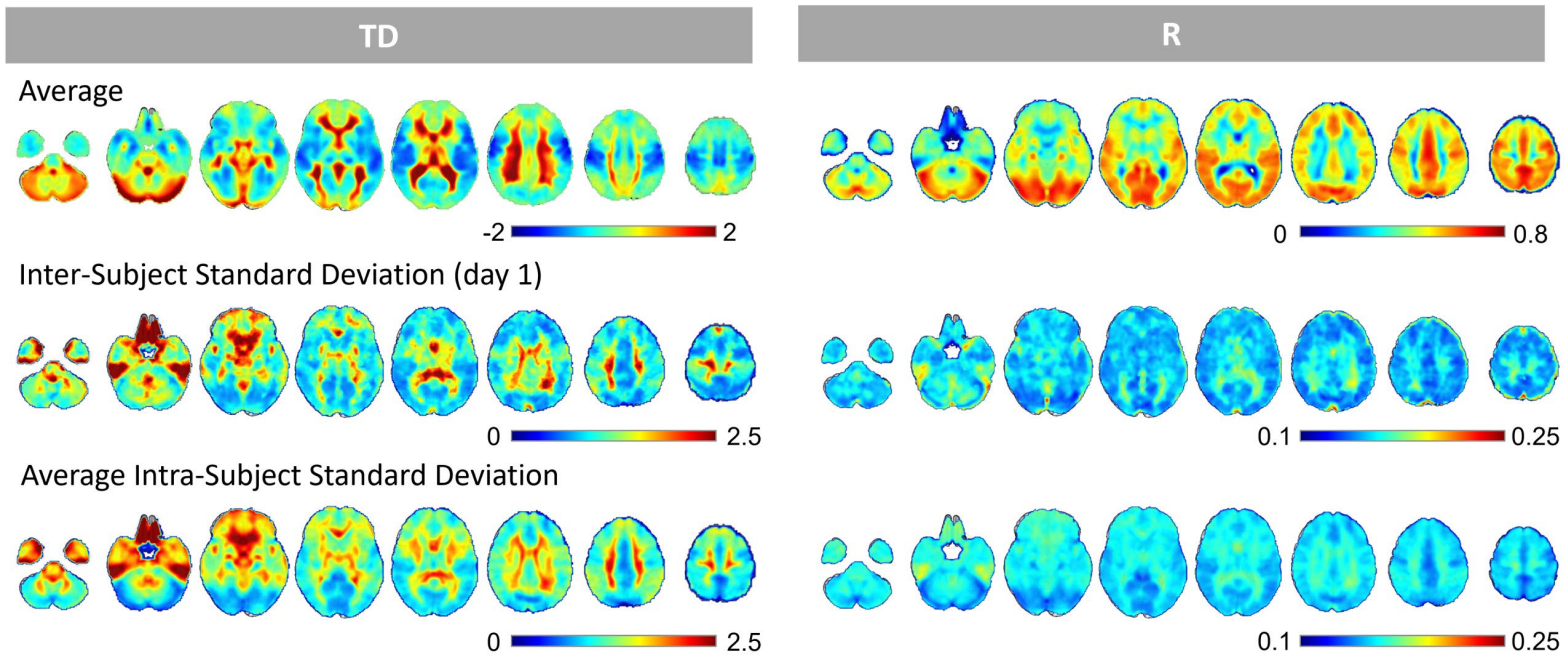
The Average and Standard Deviation Maps of the Time Delay and Correlation. The upper rows show the average time delay (TD) and correlation coefficient (R) images. The middle rows are the inter-subject standard deviation maps of TD and R (day 1). The bottom rows are the intra-subject standard deviation maps of TD and R from 26 windows x 4 runs, averaged across 50 subjects. TD and R standard deviation maps showed a similar pattern in the gray matter. The areas with higher correlation (average R) tended to show smaller standard deviations of the TD and R within the gray matter.

The average and inter- and intra-subject standard deviation maps of TD and R are shown in Figure 2 and Supplementary Figure S1. The average TD images showed a spatiotemporal pattern related to the perfusion TD, as in previous studies (Lv et al., 2013; Amemiya et al., 2014, 2016, 2022; Christen et al., 2015; Siegel et al., 2016; Tong et al., 2017; Nishida et al., 2019; Yao et al., 2021), while the correlation R images showed a positive correlation across the gray matter that was relatively small in the basal frontal areas where the susceptibility effect is considered larger. The correlation coefficients between the intra-subject standard deviation map of TD and R were 0.69 ± 0.06 (*p* < 0.001, *t* = 53.0). The correlation coefficients between the mean R and the intra-subject standard deviation maps of TD and R were TD, −0.47 ± 0.08 (*p* < 0.001, *t* = −35.7); R, −0.07 ± 0.12 (*p* < 0.001, *t* = −4.15), respectively.

### 4.2. ICA of the time delay and correlation maps of the global signal component

#### 4.2.1. Multi-subject datasets

**Figure 3:**
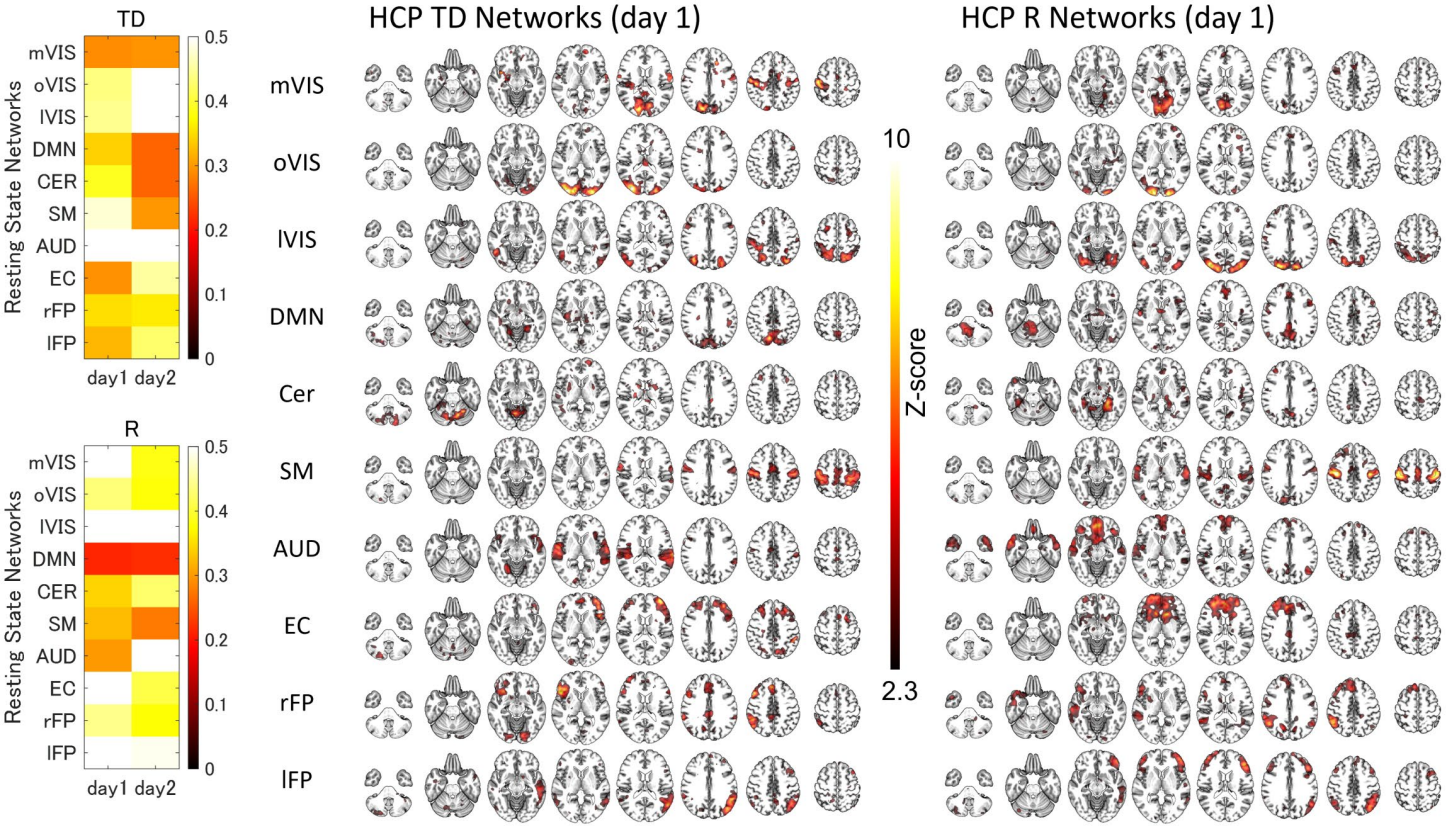
The Similarity between the Resting-State Networks and the Networks of the Inter-Individual Variability of the Time Delay and Correlation. The spatial correlation between the global signal networks (time delay [TD] and correlation [R]) and resting-state networks (RSNs) obtained from the same dataset. AUD, auditory; Cer, cerebellum; DMN, default mode network; EC, executive control; HCP, human connectome project; lFP, left frontoparietal; lVIS, lateral visual; mVIS, medial visual; rFP, right frontoparietal; oVIS, occipital visual; SM, sensorimotor.

**Figure 4:**
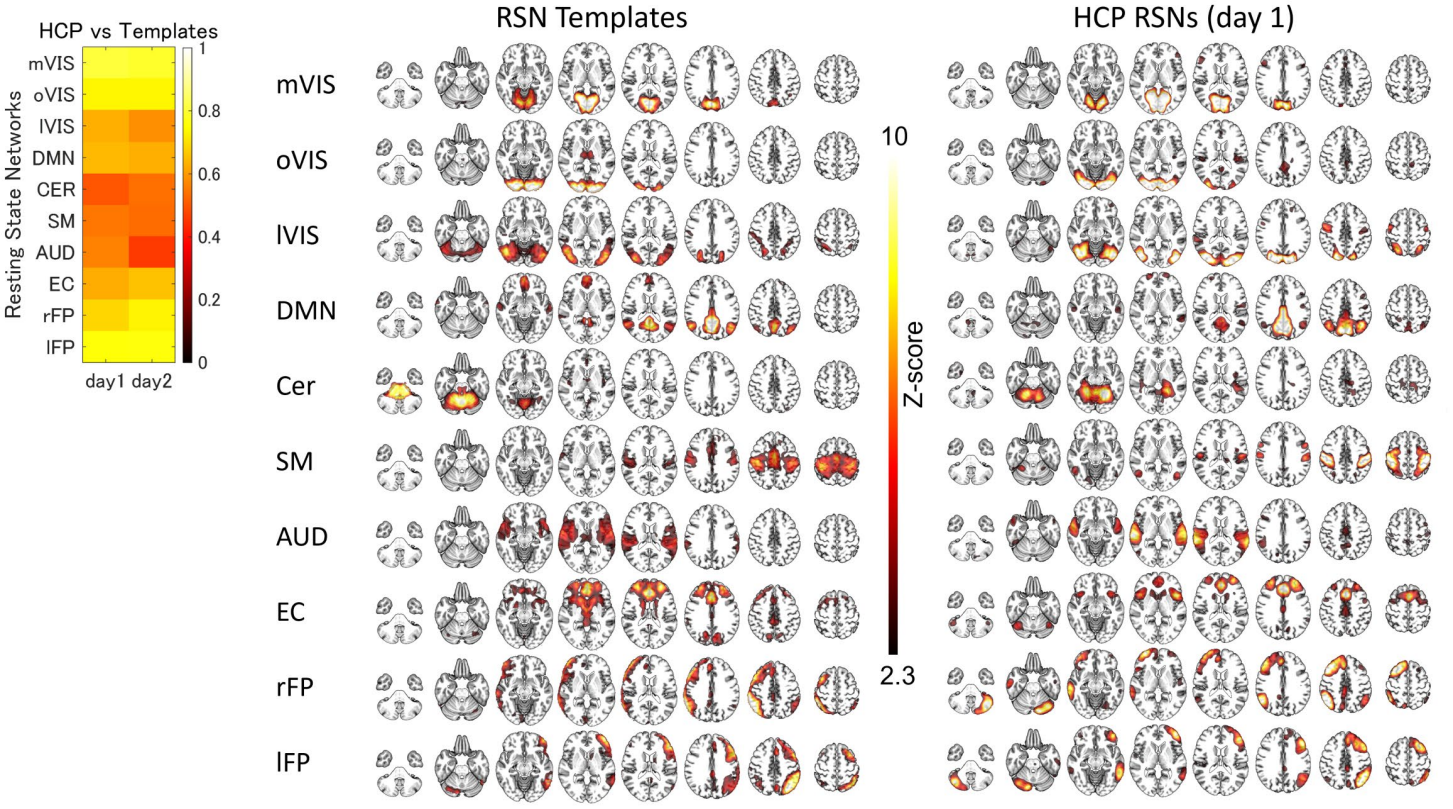
The Similarity between the HCP Resting-State Networks and Templates. The spatial correlation between the resting state network (RSN) templates and RSN maps obtained by applying spatial independent component analysis to human connectome project (HCP) datasets. AUD, auditory; Cer, cerebellum; DMN, default mode network; EC, executive control; lFP, left frontoparietal; lVIS, lateral visual; mVIS, medial visual; oVIS, occipital visual; rFP, right frontoparietal; SM, sensorimotor.

For each of the 10 RSNs (acquired from each group dataset), similar ICs were identified by applying spatial ICA to the global mean signal TD and R maps (Figure 3; Supplementary Figure S2; Supplementary Table S1). The maximum correlation coefficient between the RSNs and TD and R components for the day 1 and day 2 datasets were TD, day 1, 0.29–0.56, day 2, 0.26–0.69; R, day 1, 0.21–0.70, day 2, 0.22–0.66, respectively (Supplementary Table S1). The correlation coefficients between the RSNs with the template RSNs were day 1, 0.50–0.81; day 2, 0.46–0.79, respectively (Figure 4; Supplementary Figure S3).

#### 4.2.2. Single-subject datasets

**Figure 5:**
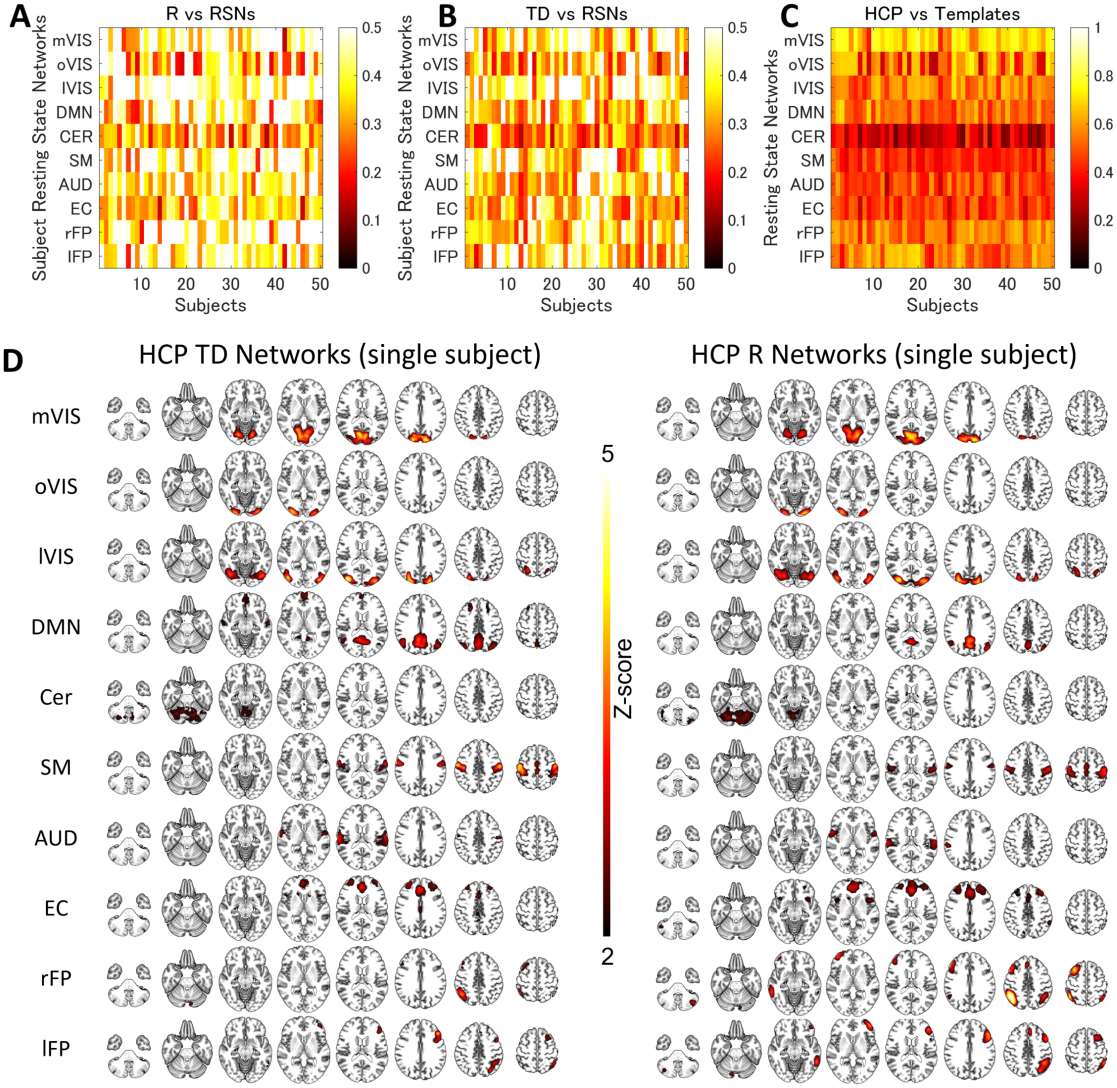
The Similarity between the Resting-State Networks and the Networks of the Intra-Individual Variability of the Time Delay and Correlation. The spatial correlation between the global signal networks (time delay [TD] and correlation [R], **A** and **B**) and resting-state networks (RSNs) obtained from each subject. **(C)** shows the correlation between the RSN templates and RSN maps obtained by applying spatial independent component analysis to each individual dataset. Each row corresponds to each subject. **(D)** shows the average single-subject spatial correlation between the global signal networks (TD and R) and resting-state networks (RSNs) obtained from the same dataset. AUD, auditory; Cer, cerebellum; DMN, default mode network; EC, executive control; lFP, left frontoparietal; lVIS, lateral visual; mVIS, medial visual; rFP, right frontoparietal; oVIS, occipital visual; SM, sensorimotor.

**Figure 6:**
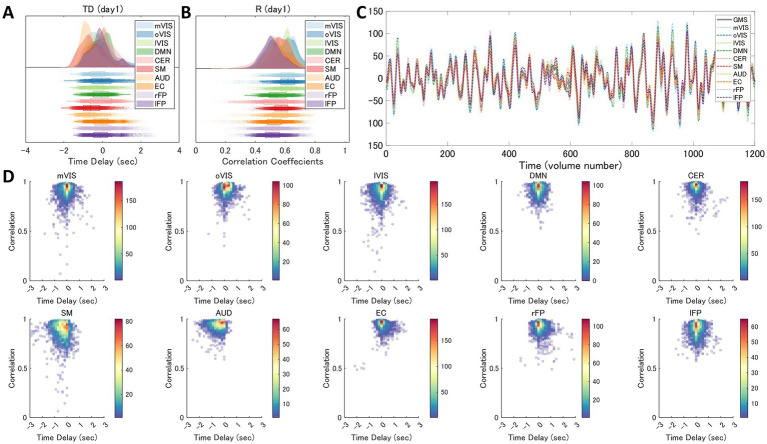
Variability of the Time Delay and Correlation in each Resting-State Network. The time delay (TD) and correlation (R) in each of the 10 resting state networks (RSNs), averaged across subjects (day 1 dataset), show that they differ in each RSN but substantially overlap with each other **(A,B)**. **(C)** shows an example RSN and global mean signal (GMS) time series from a single patient’s single run. 2D histograms **(D)** show how measured time delay (x-axis) and Pearson’s correlation between the GMS and each RSN time series (y-axis) vary within and across subjects in each RSN (pooled data of day 1 dataset comprised of 26 windows x 2 runs x 50 subjects). AUD, auditory; Cer, cerebellum; DMN, default mode network; EC, executive control; lFP, left frontoparietal; lVIS, lateral visual; mVIS, medial visual; rFP, right frontoparietal; oVIS, occipital visual; SM, sensorimotor.

**Figure 7:**
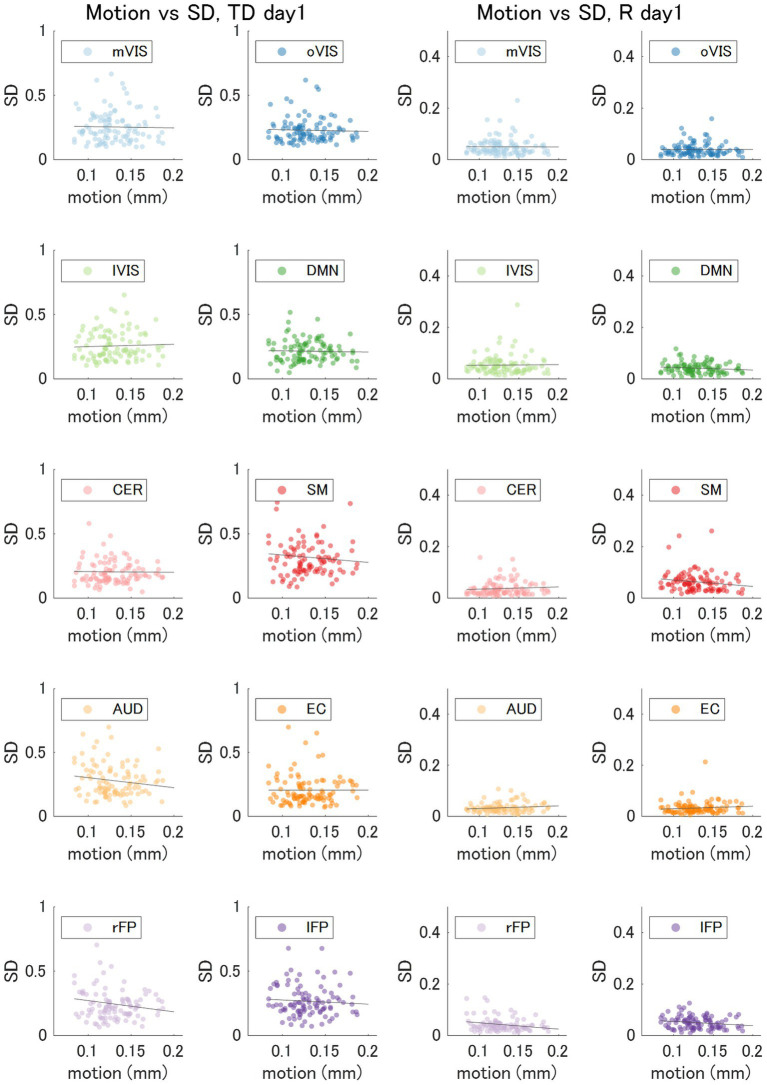
Variability of the Time Delay and Correlation versus Motion. The motion index and standard deviation of the average time delay (TD) and correlation (R) in each of the 10 resting state networks (day 1 dataset; 2 runs x 50 subjects) showed no significant correlation. AUD, auditory; Cer, cerebellum; DMN, default mode network; EC, executive control; lFP, left frontoparietal; lVIS, lateral visual; mVIS, medial visual; rFP, right frontoparietal; oVIS, occipital visual; SD, standard deviation; SM, sensorimotor.

For each of the 10 RSNs (acquired from each individual dataset), similar ICs were identified by applying spatial ICA to the global mean signal TD and R maps (Figure 5; Supplementary Table S1). The maximum correlation coefficient between the RSNs and TD and R components were all statistically significant (*p* < 0.001, TD, *t* = 16.0–23.1; R, *t* = 16.3–25.9); TD, visual (medial), 0.43 ± 0.14; visual (occipital), 0.35 ± 0.13; visual (lateral), 0.45 ± 0.13; default mode, 0.36 ± 0.11; cerebellum, 0.27 ± 0.08; sensorimotor, 0.43 ± 0.15; auditory, 0.37 ± 0.12; executive control, 0.33 ± 0.10; right frontoparietal, 0.38 ± 0.10; left frontoparietal 0.38 ± 0.11; R, visual (medial), 0.49 ± 0.15; visual (occipital), 0.38 ± 0.15; visual (lateral), 0.51 ± 0.13; default mode, 0.43 ± 0.14; cerebellum, 0.32 ± 0.10; sensorimotor, 0.46 ± 0.14; auditory, 0.41 ± 0.11; executive control, 0.36 ± 0.09; right frontoparietal, 0.48 ± 0.14; left frontoparietal 0.44 ± 0.13 (Supplementary Table S1). Figure 6 shows how the TD and correlation between the RSNs’ average signal and the global mean signal vary in each RSN (cumulative plot of 26 windows × 2 runs × 50 subjects’ data). The correlation coefficient between the RSNs with the template RSNs were all statistically significant (*p* < 0.001, *t* = 19.7–45.0); visual (medial), 0.69 ± 0.09; visual (occipital), 0.55 ± 0.15; visual (lateral), 0.56 ± 0.08; default mode, 0.53 ± 0.08; cerebellum, 0.30 ± 0.09; sensorimotor, 0.43 ± 0.06; auditory, 0.47 ± 0.08; executive control, 0.46 ± 0.10; right frontoparietal, 0.56 ± 0.08; left frontoparietal 0.56 ± 0.09, respectively. There was no significant positive correlation between the motion index and the intra-subject standard deviation of the average TD and R measurement in each RSN (TD, −0.085 ± 0.061; R, −0.11 ± 0.12, Figure 7; Supplementary Figure S5; Supplementary Table S2).

## 5. Discussion

In this study, we examined the spatiotemporal patterns of the global signal by taking advantage of the inter- and intra-subject variations of the local signal time lag and temporal correlation to understand the mechanism giving rise to the rsfMRI spatiotemporal organization, including the RSN synchronization. Consistent with the hypothesis, the ICAs showed that the variations of the TD and R, which have consistently been proven to represent local hemodynamic status (Lv et al., 2013; Amemiya et al., 2014, 2016, 2020, 2022; Christen et al., 2015; Donahue et al., 2016; Siegel et al., 2016; Khalil et al., 2017; Tong et al., 2017; Nishida et al., 2019; Yao et al., 2021) occur in the form of the spatial patterns equivalent to the RSNs. Specifically, while the group data ICA showed that the inter-subject variation of the local hemodynamics or the average hemodynamic response function organizes the RSN patterns, the intra-subject analysis revealed that a similar variation occurs within subjects across time. Despite the differences in strategy, all these results are in accord with the previous studies based on the average time lag or hemodynamic response function, further providing evidence that the variations in the local hemodynamics can be the direct source of the RSN spatial patterns (Tong et al., 2015; Amemiya et al., 2019; Chen et al., 2020).

For the intra-subject variation of the time lag, as described in the theory section, not only the changes in the local hemodynamic response functions themselves but also the changes in the power spectrum of the stimulus function (i.e., the prominence of each frequency range of stimuli) can cause changes in the time lag of the two time series generated by different hemodynamic response functions. In either case, this implies that a single set of global phenomena can cause local time lag variation, which is dependent on the average local time lag that is closely related to the hemodynamic status or hemodynamic response functions (Lv et al., 2013; Amemiya et al., 2014, 2016, 2022; Christen et al., 2015; Donahue et al., 2016; Siegel et al., 2016; Tong et al., 2017; Nishida et al., 2019; Yao et al., 2021). Such theoretical findings are well in accord with the results that not only the average time lag but also the time lag variations occur in the structured form of the RSNs. As was also consistent with the hypothesis, the analysis of the temporal correlation R also showed a similar temporal variation in the form of the RSNs.

Although the interpretation and conclusion are not necessarily the same, these results also have some points in agreement with those of the previous studies examining the rsfMRI signal time lag by applying principal component analysis or ICA. Those studies found that the whole brain rsfMRI signal time lag can be decomposed into multiple (three or eight) spatiotemporal components somehow related to the RSNs spatial patterns (Mitra et al., 2015; Amemiya et al., 2016; Bolt et al., 2022). In interpreting such findings, it is important to note that, unlike task fMRI, the unavailability of knowledge about the stimuli poses a difficulty to the rsfMRI. Other than respiratory- and cardiac-related physiological recordings, the spatiotemporal distribution of the data is the only clue that distinguishes if the source is neural or not. It is, therefore, often difficult to know the source of each observation in rsfMRI. However, with further information, such as the high consistency of the spatiotemporal patterns of the rs-fMRI signal time lag (Amemiya et al., 2019, 2020) suggesting its hemodynamic origin (Amemiya et al., 2020), we considered it more reasonable to conclude that the multiplicity of the dimensions of the rsfMRI time lag structures results from the variability of the hemodynamics in each region. In this study, we thus hypothesized that inter- and intra-subject variability of the hemodynamic response functions giving rise to the temporal variability of the time lag is the direct source of the RSNs’ spatial patterns. The dimensionality of the ICA was, therefore, set to 30, which is a common number for investigating the RSNs (Beckmann et al., 2005; Tong et al., 2015). Although this was empirically determined, the results showed that the hypothesis was true. Even asymmetric networks, like frontotemporal networks, whose average time lag and temporal correlation distributions were highly overlapped (Figures 6A,B), were decomposed into two networks (Figures 3, 5). That any single global phenomenon inducing the BOLD responses can lead to the RSN representation is an even more parsimonious description of the spatiotemporal organization of the rsfMRI signals compared with the previous global signal models (Mitra et al., 2015; Bolt et al., 2022) let alone the classic view of the RSNs assuming network-specific regulation for the local coactivation of the neurons.

The assumed sources of the global and simultaneous phenomenon include respiratory- and cardiac-related factors such as variations in heart rate (Chang et al., 2009) and blood pressure (Zhang et al., 2000), respiration volume per time (Birn et al., 2006, 2008) and partial pressure of end-tidal carbon dioxide (Wise et al., 2004). All these interrelated factors are known to alter the cerebral blood flow or blood volume systematically, thereby affecting the BOLD signal in the low-frequency range of interest. Although the primary source of the global BOLD signal is likely the physiological noise (Liu et al., 2017), it could also have some neural components, as was suggested in the electroencephalographic work in humans and microelectrode recordings in anesthetized monkeys (Leopold et al., 2003; He et al., 2008; Scholvinck et al., 2010; Liu et al., 2018).

The variation of the TD and R in the form of RSNs shown in the intra-subject analysis not only explains how the classic RSNs can arise from a global phenomenon but could also explain the variability in the inter-network correlations over time as the results of the temporal changes in the local hemodynamics and/or in the power spectrum of the global events. Such an observation adds further challenges to the studies dealing with the dynamic or time-varying functional connectivity using rsfMRI (Preti et al., 2017) that have seen rapidly growing interest in recent years as a clinical diagnostic marker of psychiatric disorders (Lurie et al., 2020).

There are some limitations and technical considerations to be taken into account when interpreting the results. Firstly, for the intra-subject analysis, we used the sliding window approach. While this is a traditional and simple method often employed in dynamic functional connectivity studies, the choice of window length is arbitrary. This is because, other than setting a lower limit to the largest wavelength to avoid artifacts (Leonardi and Van De Ville, 2015), there is no clear indication of the window size to achieve the best trade-off between the reliable computation of correlation and detection of the temporal variations of interest (Preti et al., 2017). Secondly, the intra-subject variation of the TD and R likely reflects the measurement errors to some extent, although no significant effect of motion was found for any RSN. As was shown in the whole-brain analysis of the mean and standard deviation maps of TD and R, the regions with smaller standard deviations tended to have a larger correlation with the global mean signal. This could be attributed to larger measurement errors in the presence of signals other than the global mean signal. However, given the fact that the variations occur not randomly but in the structured form of the RSNs, they seem to be related to physiological phenomena. Thirdly, although it has empirically been shown that the rsfMRI signal TD relative to the global mean signal corresponds to the perfusion delay, it is less clear if the contribution from the local signals is also negligible in the evaluation of the variability of TD and R. Indeed, it is difficult to entirely deny the possibility that the correlation between the RSN and TD/R ICs is enhanced by the fact that signals within each RSN covary. However, as far as we examined in a simulation study, the correlation is less likely to reflect an artifact (see Supplementary material). Finally, as for the interpretation of the dynamic changes in the time lag, it might be difficult to completely exclude the possibility that the changes are related to the network-specific neural activity. However, it is important to note that there is no evidence supporting such a view, which is in contrast to the highly coherent findings suggesting its hemodynamic origin for the average as well as the inter- and intra-subject variability of the time lag in a series of studies (Lv et al., 2013; Amemiya et al., 2014, 2016, 2020, 2022; Christen et al., 2015; Donahue et al., 2016; Siegel et al., 2016; Khalil et al., 2017; Tong et al., 2017; Nishida et al., 2019; Yao et al., 2021). We thus consider it more reasonable to attribute it to the hemodynamic changes.

## 6. Conclusion

In summary, by taking advantage of the inter- and intra-subject variations of the local signal time lag and correlation, we examined the spatiotemporal patterns of the global signal, which have consistently been proven to represent local hemodynamic status and found that they organize the spatial patterns equivalent to the RSNs. These data not only indicate that any single global phenomenon inducing hemodynamic responses can result in the RSN representation but also explain how the regional variations in the hemodynamics, combined with the changes in the power spectrum of the global events, can lead to the observations. While the findings pose challenges to the interpretation of rsfMRI studies, they further support the view that rsfMRI can offer detailed information related to global neurophysiological phenomena as well as local hemodynamics that would have great potential as biomarkers.

## Data availability statement

Publicly available datasets were analyzed in this study. This data can be found at: https://www.humanconnectome.org/.

## Author contributions

SA contributed to conception and design of the study, performed the statistical analysis, and wrote the first draft of the manuscript. All authors contributed to the article and approved the submitted version.

## Funding

This work was supported by JSPS Grant-in-Aid for Scientific Research (C) 21K07720 and 18K07707 and the 14th Shiseido Female Researcher Science Grant to SA.

## Conflict of interest

The authors declare that the research was conducted in the absence of any commercial or financial relationships that could be construed as a potential conflict of interest.

## Publisher’s note

All claims expressed in this article are solely those of the authors and do not necessarily represent those of their affiliated organizations, or those of the publisher, the editors and the reviewers. Any product that may be evaluated in this article, or claim that may be made by its manufacturer, is not guaranteed or endorsed by the publisher.

## Footnotes

^1^https://www.humanconnectome.org/study/hcp-young-adult
