# Supplementary material for: Resting-state networks representation of the global phenomena
Source: Front Neurosci. 2023 Aug 17;17:1220848. doi: 10.3389/fnins.2023.1220848 (PMC10469869; doi:10.3389/fnins.2023.1220848)
Supplement: Supplementary file 1 [file Data_Sheet_1.docx]

*Resting-State Networks Representation of the Global Phenomena*

Shiori Amemiya^*^, Hidemasa Takao, Shouhei Hanaoka, Osamu Abe

*** Correspondence:** Shiori Amemiya: amemiya-tky@umin.ac.jp; [mamemiya@g.ecc.u-tokyo.ac.jp](mailto:mamemiya@g.ecc.u-tokyo.ac.jp)

# Supplementary Figures

## Figure S1.
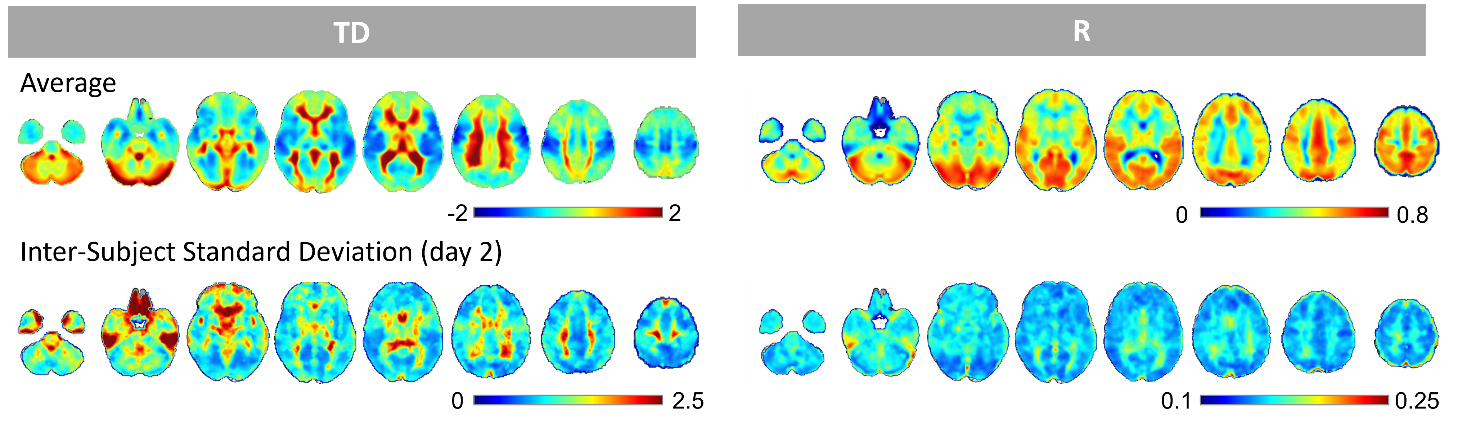
The Average and Standard Deviation Maps of the Time Delay and Correlation (day 2).

**Supplementary Figure 1.** The upper rows show the average time delay (TD) and correlation coefficient (R) images. The middle rows are the inter-subject standard deviation maps of TD and R (day 2). The bottom rows are the intra-subject standard deviation maps of TD and R from 26 windows x 4 runs, averaged across 50 subjects. TD and R standard deviation maps showed a similar pattern in the gray matter.

## Figure S2.
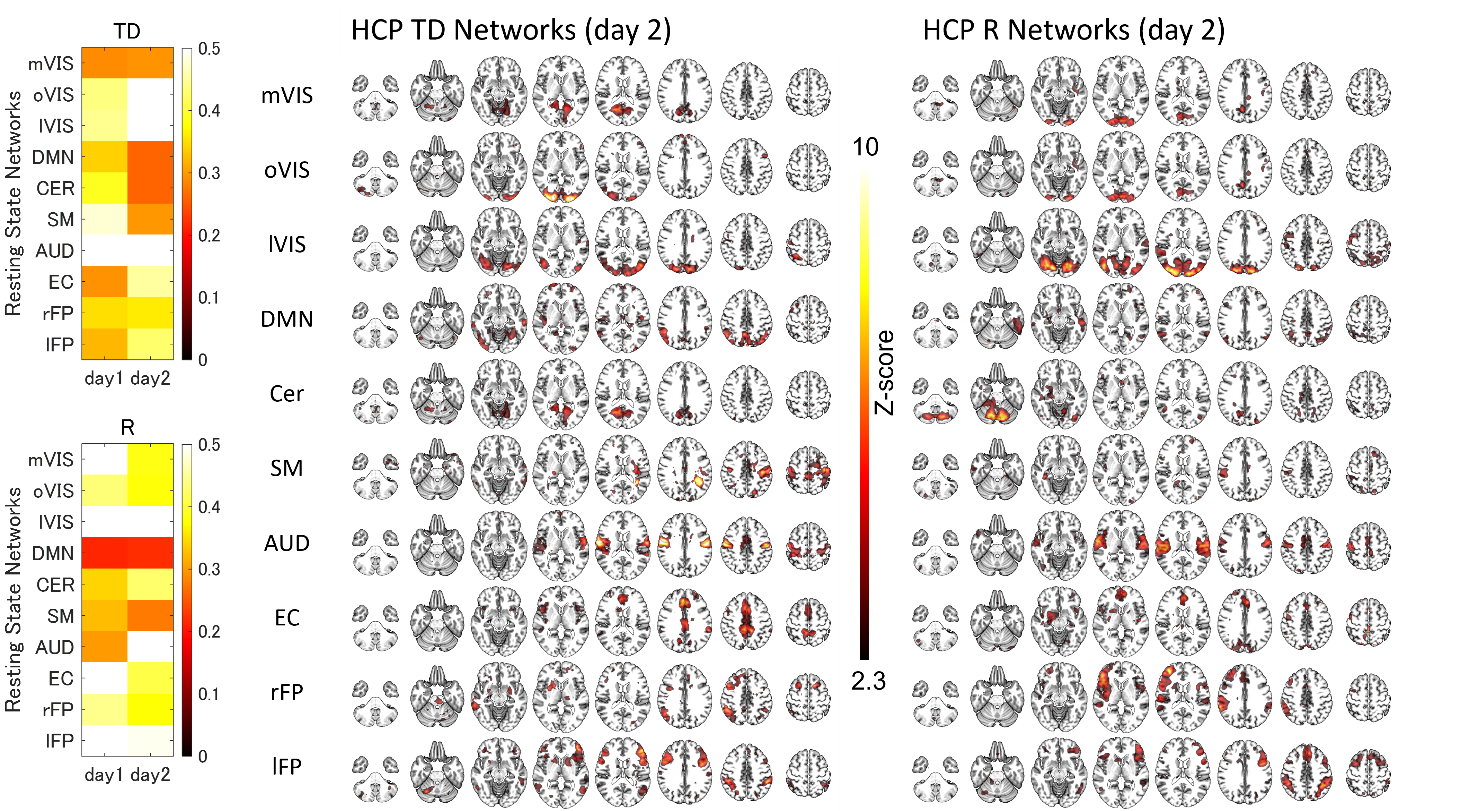
The Similarity between the Resting-State Networks and the Networks of the Inter-individual Variability of the Time Delay and Correlation (day 2).

**Supplementary Figure 2.** The spatial correlation between the global signal networks (time delay [TD] and correlation [R]) and resting-state networks (RSNs) obtained from the same dataset. *AUD, auditory; Cer, cerebellum; DMN, default mode network; EC, executive control; lFP, left frontoparietal; lVIS, lateral visual; mVIS, medial visual; oVIS, occipital visual; rFP, right frontoparietal; SM, sensorimotor.*

##
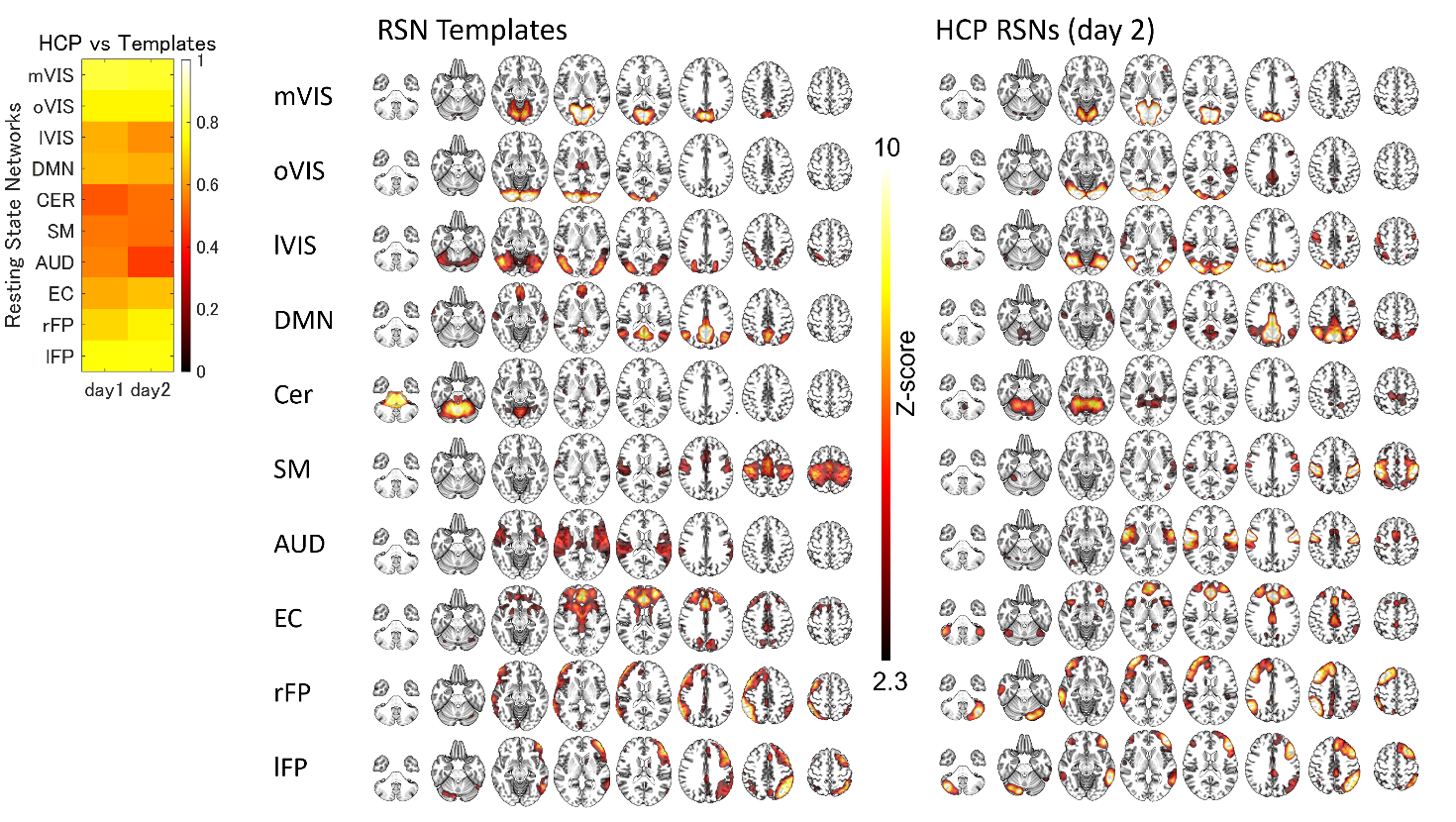
Figure S3. The Similarity between the HCP Resting-State Networks and Templates (day 2).

**Supplementary Figure 3.** The spatial correlation between the RSN templates and RSN maps obtained by applying spatial independent component analysis to human connectome project (HCP) datasets. *AUD, auditory; Cer, cerebellum; DMN, default mode network; EC, executive control; lFP, left frontoparietal; lVIS, lateral visual; mVIS, medial visual; oVIS, occipital visual; rFP, right frontoparietal; SM, sensorimotor.*

## Figure S4. Variability of the Time Delay and Correlation in each Resting-State Network (day 2).


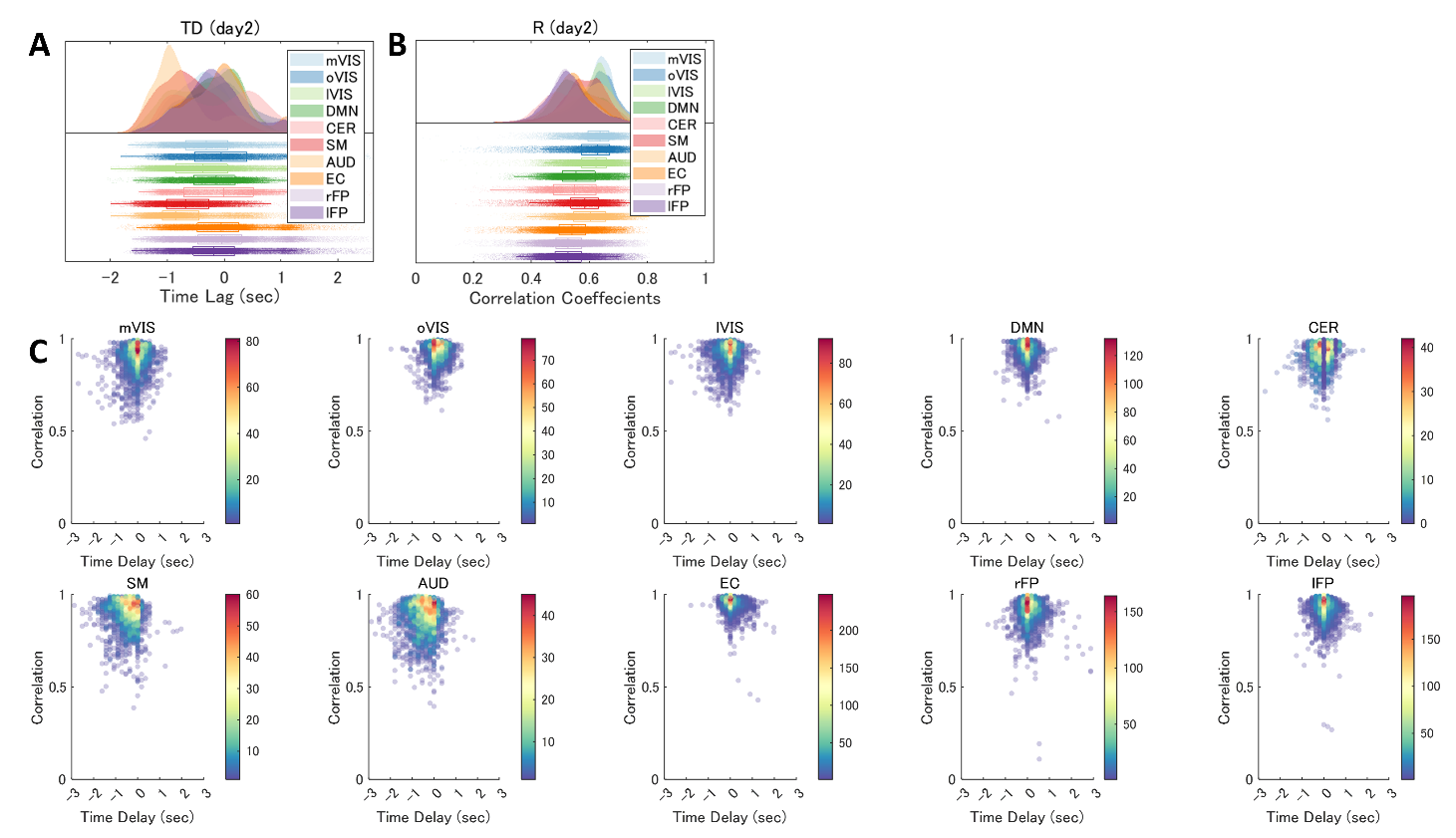


**Supplementary Figure 4.** The time delay (TD) and correlation (R) in each of the 10 resting state networks (RSNs), averaged across subjects (day 2 dataset), show that they differ in each RSN but substantially overlap with each other (A, B). 2D histograms (C) show how measured time delay (x-axis) and Pearson's correlation between the global mean signal and each RSN time series (y-axis) vary within and across subjects in each RSN (pooled data of day 2 dataset comprised of 26 windows x 2 runs x 50 subjects). *AUD, auditory; Cer, cerebellum; DMN, default mode network; EC, executive control; lFP, left frontoparietal; lVIS, lateral visual; mVIS, medial visual; oVIS, occipital visual; rFP, right frontoparietal; SM, sensorimotor.*

## Figure S5. Variability of the Time Delay and Correlation versus Motion (day 2).


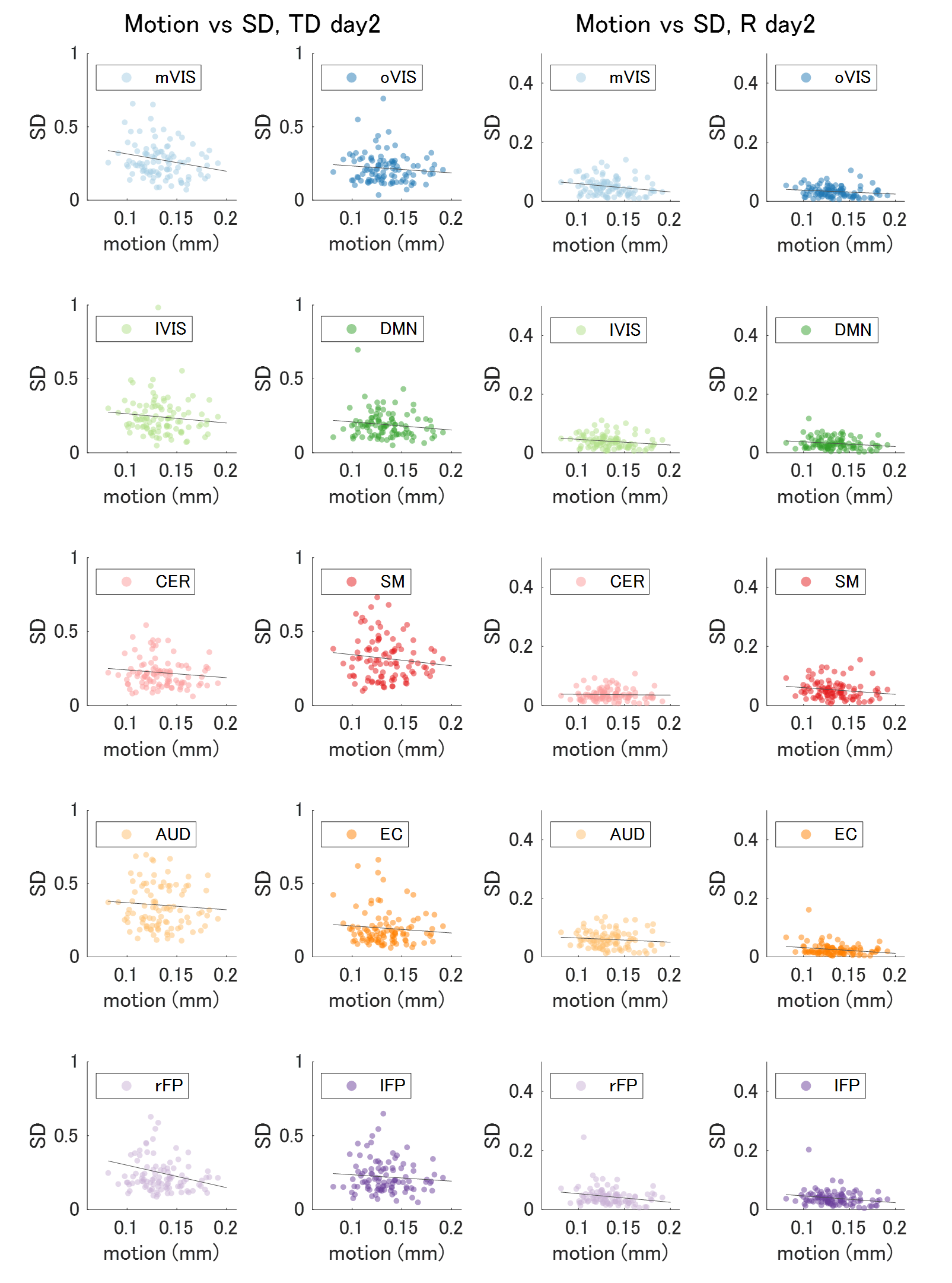


**Supplementary Figure 5.** The motion index and standard deviation of the time delay (TD) and correlation (R) in each of the 10 resting state networks (RSNs) (day 2 dataset; 2 runs x 50 subjects) showed no significant correlation. *AUD, auditory; Cer, cerebellum; DMN, default mode network; EC, executive control; lFP, left frontoparietal; lVIS, lateral visual; mVIS, medial visual; rFP, right frontoparietal; oVIS, occipital visual; SD, standard deviation; SM, sensorimotor.*

# Supplementary Tables

## Table S1. The Similarity between the Resting-State Networks and Time Delay/Correlation Components

|  | **Intra-Subject Analysis** | | | | **Inter-Subject Analysis** | | | |
| --- | --- | --- | --- | --- | --- | --- | --- | --- |
|  | **TD** | | **R** | | **TD** | | **R** | |
|  | **Day 1** | **Day 2** | **Day 1** | **Day 2** | **Mean** | **SD** | **Mean** | **SD** |
| **mVIS** | 0.29 | 0.30 | 0.58 | 0.38 | 0.43 | 0.14 | 0.49 | 0.15 |
| **lVIS** | 0.44 | 0.55s | 0.43 | 0.38 | 0.35 | 0.13 | 0.38 | 0.15 |
| **oVIS** | 0.44 | 0.65 | 0.70 | 0.66 | 0.45 | 0.13 | 0.51 | 0.13 |
| **DMN** | 0.34 | 0.26 | 0.21 | 0.22 | 0.36 | 0.11 | 0.43 | 0.14 |
| **Cer** | 0.39 | 0.26 | 0.34 | 0.43 | 0.27 | 0.08 | 0.32 | 0.10 |
| **SM** | 0.48 | 0.30 | 0.32 | 0.28 | 0.43 | 0.15 | 0.46 | 0.14 |
| **AUD** | 0.56 | 0.69 | 0.30 | 0.63 | 0.37 | 0.12 | 0.41 | 0.11 |
| **EC** | 0.29 | 0.45 | 0.51 | 0.41 | 0.33 | 0.10 | 0.36 | 0.09 |
| **rFP** | 0.35 | 0.36 | 0.44 | 0.38 | 0.38 | 0.10 | 0.48 | 0.14 |
| **lFP** | 0.32 | 0.43 | 0.57 | 0.49 | 0.38 | 0.11 | 0.44 | 0.13 |

*AUD, auditory; Cer, cerebellum; DMN, default mode network; EC, executive control; lFP, left frontoparietal; lVIS, lateral visual; mVIS, medial visual; R, Pearson’s correlation coefficient; rFP, right frontoparietal; oVIS, occipital visual; RSNs, resting state networks; SD, standard deviation; SM, sensorimotor; TD, time delay.*

## Table S2. The Correlation Coefficient between the Intra-Subject Variability of the Time Delay/Correlation and Motion Index.

|  | **TD** | | **R** | |
| --- | --- | --- | --- | --- |
|  | **Day 1** | **Day 2** | **Day 1** | **Day 2** |
| **mVIS** | -0.003 | -0.223 | -0.020 | -0.190 |
| **lVIS** | 0.000 | -0.162 | -0.033 | -0.108 |
| **oVIS** | 0.014 | -0.195 | 0.041 | -0.112 |
| **DMN** | -0.126 | -0.186 | -0.028 | -0.138 |
| **Cer** | 0.075 | -0.031 | -0.013 | -0.106 |
| **SM** | -0.157 | -0.170 | -0.080 | -0.113 |
| **AUD** | 0.133 | -0.108 | -0.154 | -0.064 |
| **EC** | 0.098 | -0.220 | 0.002 | -0.093 |
| **rFP** | -0.233 | -0.219 | -0.148 | -0.189 |
| **lFP** | -0.174 | -0.214 | -0.068 | -0.089 |

*AUD, auditory; Cer, cerebellum; DMN, default mode network; EC, executive control; lFP, left frontoparietal; lVIS, lateral visual; mVIS, medial visual; R, Pearson’s correlation coefficient; rFP, right frontoparietal; oVIS, occipital visual; RSNs, resting state networks; SM, sensorimotor; TD, time delay.*

# Supplementary Simulation Study

As described in the introduction, it has empirically been shown that the rsfMRI signal time delay relative to the global mean signal corresponds to the perfusion delay rather than the RSNs structure (Lv et al., 2013; Amemiya et al., 2014; Amemiya et al., 2016; Tong et al., 2017; Amemiya et al., 2022; Amemiya et al., 2023). This indicates that the effect from the local signals that are specific to each RSN is practically negligible in this computation. However, it is less clear if the contribution from the local signals is also negligible in the evaluation of the variability of TD and R. Therefore, we performed a simulation study to examine if the correlation between the TD/R ICs and RSNs arise as an artifact.

## Methods

Using white Gaussian noise, four local signal time series, a pair of global signal time series, and 100 x 100 noise time series with a mean of 0 and lasting for 12,000 time points were generated. Data subjected to the following analyses were prepared by summing up these signals (i.e., four local signals, two global signals, and a noise for each time series) and were arranged on a 100 × 100 grid, with the magnitude of the local signals S1, S2, S3, and S4 varied progressively from the four vertexes to the contralateral vertexes via the center of the square along the diagonal axes from 0 to 1 in equal increments, respectively. The global signals and noise time series were scaled to have a standard deviation of 2 and 0.2 of the S1–S4 signal component, respectively. The magnitude and time lag of the global signals varied from 0 to 1 and - 6 to 6 sec with a random Gaussian distribution. The data were up-sampled to a resolution of 0.072 sec (1/10 TR) and subjected to bandpass filtering at 0.01–0.1 Hz.

Four runs of datasets were concatenated (100 rows x 100 columns x 120,000 time points x 4 runs) and were subjected to an ICA using the FastICA package (Hyvärinen, 1999) with tanh non-linearity and a symmetric decorrelation approach with the dimensionality set to 11. Within subject TD/R variability was examined using a sliding-window approach (window length/step size = 20,000/4,000 volumes, 26 maps/run x 4 runs) as in the single-subject analysis in the main study. TD/R maps (26 x 4) generated from the sliding-window analysis, using the average of the 100 x 100 time series as the reference signal, were similarly subjected to an ICA, respectively.

ICs from the original dataset included three structured components related to the arrangement of the four signals on the grid. These were identified as ICs having a Pearson’s correlation coefficient larger than 0.4 with the ICs obtained from the datasets composed only of the four signals S1–S4. For each of the structured components from the original time series, a single IC that gives the largest correlation with the structured component was identified from among the ICs of the TD and R maps, respectively. The same analysis was performed for 50 times.

## Results

Figure S6 D–F shows example ICs obtained from the original dataset (corresponding to RSNs in the main study). Those ICs have diagonal, horizontal, or perpendicular gradations corresponding to those of S1–S4 (Figure S6 A–C). Figure S6 G–L shows ICs from the TD (G, H, I) and R (J, K, L) maps giving the largest correlation with the ICs from the original time series D, E, and F, respectively. The correlation between the two datasets across the 50 trials were (TD, 0.16 ± 0.07; R, 0.13 ± 0.06). TD/R ICs also visually showed random patterns (G–L).

**Reference:** Hyvärinen, A. (1999). Fast and robust fixed-point algorithms for independent component analysis. *IEEE transactions on Neural Networks* 10(3)**,** 626-634.

##
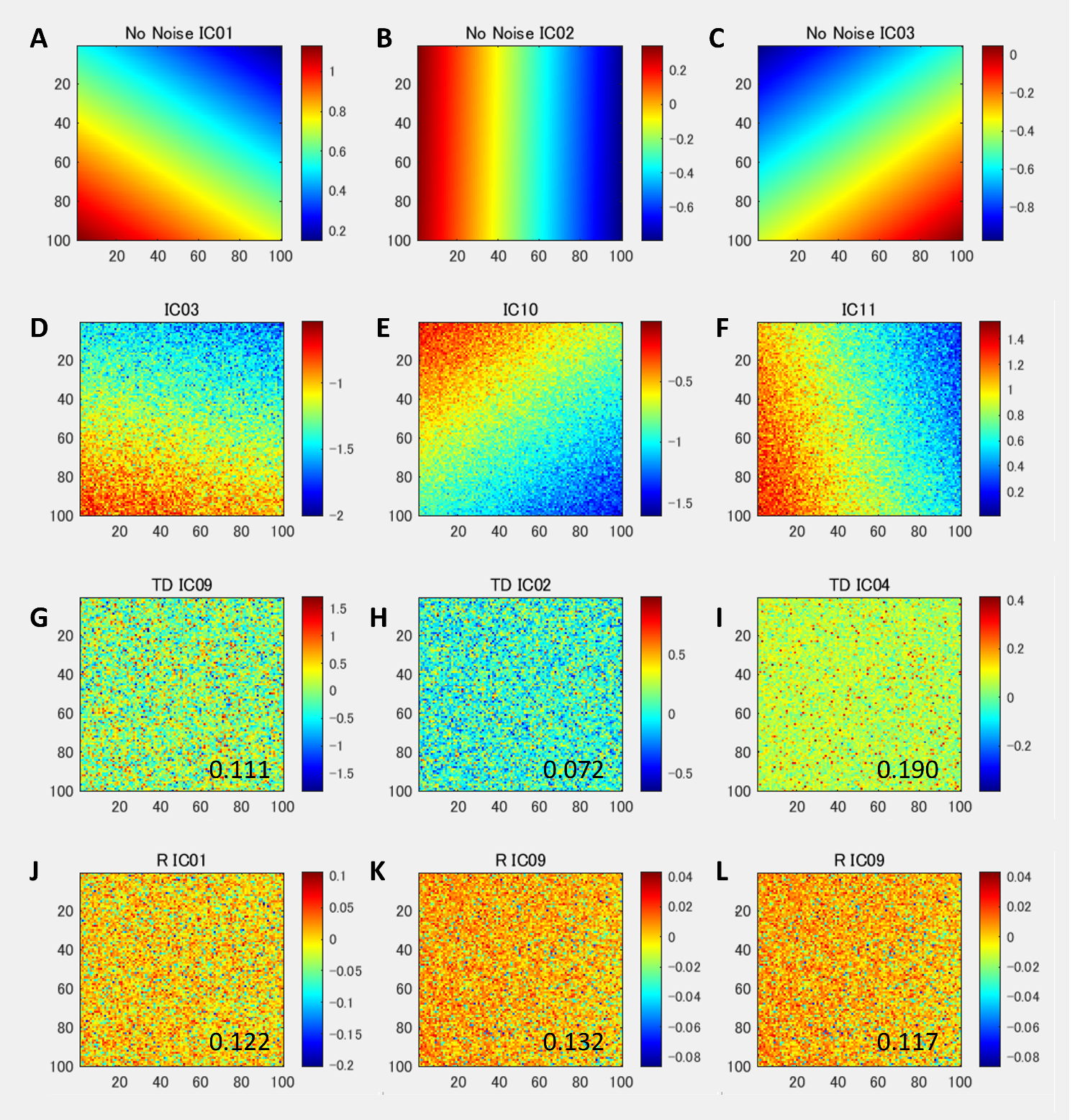
Figure S6. Effect of the Local Signals on the TD/R Variance Evaluation.

The numbers indicate the Pearson’s correlation coefficient between each time delay (TD) or R (Pearson’s correlation coefficient) component (G–L) and the corresponding independent component (IC) of the original dataset (D, E, and F), respectively. G and J correspond to D, H and K correspond to E, while I and L correspond to F, respectively.
